# Data of risk analysis management in university campuses

**DOI:** 10.1186/s13104-020-05397-4

**Published:** 2020-12-09

**Authors:** Alireza Dehdashti, Farin Fatemi, Muhammadreza Janati, Fatemeh Asadi, Marzieh Belji Kangarloo

**Affiliations:** 1 Research Center for Health Sciences and Technologies, Semnan University of Medical Sciences, Semnan, Iran; 2 Student Research Committee, Semnan University of Medical Siences, Semnan, Iran

**Keywords:** Hazards, Risk assessment, Academic settings

## Abstract

**Objectives:**

This data paper aims to provide the data set of a practical method to health, safety, and environmental risk assessment to assess and rank potential threats/hazards and to prevent and decrease the accidents and harmful consequences at an academic setting. Descriptive data on type of hazards, places, and persons at risk were collected. Quantitative data on risk probability and severity of identified hazards were determined. Additionally, the descriptive statistics and analytical tests were applied to create a concise perspective on health, safety and environmental hazards/threats situation in research location under study. The dataset further provides information on the prioritization of determined risks according to the relevant scores and levels for doing the relevant control measures to remove and mitigate the related risks.

**Data description:**

This paper provides data of comprehensive risk assessment of health, safety and, environmental hazards of academic setting. For each identified hazard, the descriptive and numeric data are available. The information about the risk level and prevention or mitigation measure related to each hazard is provided. Additionally, the statistical tests are applied for determining the relations among the variables under study. The data and methodology on risk assessment in this article may be used to manage variety of risks in higher education institutions.

## Objective

Risk assessment is the process of evaluating risks to persons’ safety and health from workplace hazards [1]. This data note aims to provide comprehensive information about the Health, Safety and Environment (HSE) risk assessment. Such these data sets are necessary for accident prevention and decrease the harmful impacts such as death, injuries and damages to structures and equipment at academic settings [2, 3]. Also, such these risk assessment results promote information sharing across the university systems about best practices to mitigate the vulnerability of hazards [4]. Corresponding mitigation measures for each campus’s highest risks is beneficial to create a University-wide relative risk ranking of all threat events and to summarize the status of campus mitigation measures [5]. Taking steps to either eliminate or to reduce risks (as far as reasonably practicable) by introducing control measures should be done in the risk management phase [6]. To achieve these goals, we developed two checklists to collect descriptive and quantitative data based on the evidence review [7, 8]. The laboratories, public offices, and peripheral areas of the academic setting were included in this study. Besides, we planned to determine the hazard mitigation strategies for each identified hazard in the assessed areas of the academic setting in the applied checklist [9]. In the current paper, we described the collected data during the HSE risk assessment in this study. A research paper was written up and published based on our data and methodology to further describe and prioritize risks in terms of varying risk impacts in the university environment [10]. These data may help researchers to assess probability, impact, and mitigation risks in detail and find a proactive approach toward risks in higher education institutions.

## Data description

This cross-sectional study was conducted from June to July 2018 at the School of Public Health, Semnan, University of Medical Sciences, Semnan, Iran. For implementing a successful HSE risk assessment, we needed to identify the potential hazards into three individual domains of health, safety, and environment in different areas of the academic setting. First, a list of hazards in three sections of health, safety, and environment was developed in a checklist. The reviewed literature on concerning recent accidents or incidents within universities was much helpful in defining the list of HSE hazards. The hazards checklist was completed via on-site walking and interviews by three trained students in the 39 locations of the academic setting. Additionally, the persons at risk including students, staff, or faculties were determined for each hazard in the assessed location. The descriptive analysis indicated that the most frequency of hazards was related to health hazards (50.3%). The safety and environmental hazards were 44%, and 5.7%, respectively.

Second, the research team analyzed the risks based on the risk matrix in ISO 31000 [8] (see Table 1). Risk analysis was done by estimating the probability and severity of risks for identified hazards. The probability of occurrence metrics was measured on a five-point scale from “not applicable” to “inevitable”. The interpretation of each scale has been mentioned in data file 1 [11]. The novelty of this study is determining the severity rate in terms of human, equipment, and institution. To estimate the effect of each identified hazard on three mentioned terms, we applied two items with a five-point score response [12]. Responses were scored and averaged to obtain an overall severity score. The severity metrics were measured on a five-point scale from “very low” to “very high”. The final risk scores were calculated from multiplying probability by severity and categorized into 3 risk levels, risks rated in the top category (red) are larger than those rated in moderate category (yellow) and low category (green).

**Table 1:** Overview of data files

Label	Name of data file	File types (file extension)	Data repository and identifier (DOI or accession number)
Data file 1	Risk assessment data	Excel file (.xlsx)	10.6084/m9.figshare.13106825.v1 [11]
Data file 2	Analytical data	SPSS files-Variables and output (.sav)	10.6084/m9.figshare.13106843.v1 [13]

Based on the results, hazards with high risk level were belonged to safety category that they were included 5.7%, required immediate mitigation measures. From all identified health, safety and environmental hazards, 6.5%, 36% and 43% were categorized in moderate risk level, respectively. These hazards need to be corrected in the near future. The rest of identified HSE hazards had acceptable risk score and categorized in low risk hazards. Therefore, the mitigation measures were recommended in appropriate to determined risk levels for identified hazards.

In data file 2, in order to create a more detailed view on health, safety and environmental hazards/threats situation, the analytical tests were applied to examine associations among the main variables under study [13]. Type of analytical tests (Chi square or Kruskal–Wallis)was used based on type of variable that is qualitative or quantitative. The most important finding is related to significant relation between the calculated risk scores and the type of hazards. The safety-related hazards indicated a statistically higher contribution to the total risk score when compared to health and environmental hazards. The descriptive analysis and providing analytical information helps to withstand and cope with the adverse effects of accidents and emergencies [14, 15].

## Limitations

Although risk matrices can indeed be very useful in risk analysis, but can mistakenly assign higher qualitative ratings to smaller risks that leading to wrong risk management decision. It means that we might have quantitatively overestimated or underestimated the risk scores for identified hazards. Probably, applying new approaches to risk matrix such as new scaling and scoring methods to risk matrix extensions depends on the location of risk assessment in future studies would minimize the mentioned concern.

The data of this study were collected in a typical higher education institution with its exclusive hazards, which may limit its interpretation of safety, health, and environmental risks. However, the authors believe that an integrated approach for obtaining data will be beneficial to compare various domains of hazards in a workplace.

## Data Availability

The data described in this Data note can be freely and openly accessed via http://figshare.com. Please see Table 1 and Refs. [11, 13] for details and links to the data.

## Abbreviation

HSE: Health, safety, environment
